# List of Reviewers

**DOI:** 10.1093/jrr/rrv082

**Published:** 2015-10-29

We would like to express our greatest appreciation to our reviewers. *Journal of Radiation Research* Vol. 56 (2015) benefited from the comments and insights from the following 287 reviewers. We sincerely thank them for their time devoted and effort which contributed to the improvement of the journal. (Reviewers who reviewed three or more manuscripts are listed in bold font.)

Adriano Garonna
Akihiro Nohtomi
Akihisa Takahashi
Akinari Yokoya
Akira Endo
Alessandro Clivio
Alexander Ivannikov
Alice Sigurdson
Anna L. Petoukhova
**Arindam Chaudhury**
**Asako Nakamura**
Asao Noda
Atsunori Yorozu
Atsushi Enomoto
Atsushi Ito
Atsushi Shibata
Atsuya Takeda
Atsuyuki Sorimachi
B. Almayahi
Bilikere Dwarakanath
**Bing Wang**
Brad Christian
C. Karger
Chang Song
Chie Kurokawa
Chihiro Kanehira
Daisuke Iizuka
David Grdina
Deborah Citrin
Dharmendra Maurya
Dr. Vijayalaxmi
Fred Mettler
Fujio Araki
Fumiaki Isohashi
Fumiya Baba
Gabor Andocs
**Gaurav Kumar**
Gavin Wang
Gen Yang
Guangming Zhou
**Guillaume Vares**
Hari Kumar Kb
Hidenobu Tachibana
Hidetaka Arimura
Hidetoshi Shimizu
Hideya Yamazaki
Hideyuki Mizuno
Hiroaki Terato
Hiroki Tanaka
Hiromichi Gomi
Hiromichi Ishiyama
Hironobu Ikehata
Hironobu Yasui
Hironori Yoshino
Hiroo Nakajima
Hiroshi Fuji
Hiroshi Harada
Hiroshi Ide
Hiroshi Igaki
Hiroshi Maezawa
Hiroshi Masuda
Hiroshi Onishi
**Hiroshi Sekine**
Hiroshi Tauchi
Hiroshi Watanabe
Hiroyuki Katoh
Hiroyuki Okamoto
Hisato Nagano
**Hitoshi Ikushima**
Hitoshi Ishikawa
Hitoshi Satoh
Ikuo Kashiwakura
Iuliana Toma-Dasu
Jacqueline Williams
James C L Chow
Janapriya Saha
Joel Greenberger
John Baker
Jun Hidema
**Jun Itami**
Kassym Zhumadilov
Katarzyna Leszczynska
Katsumasa Nakamura
Katsumi Imaida
Katsumi Tsujioka
Katsunori Yogo
Katsuya Maebayashi
Katsuyuki Karasawa
Katsuyuki Sakanaka
Kayoko Tsujino
Kazuhiro Daino
Kazushige Hayakawa
Kei Shibuya
**Keiichi Akahane**
Keiichi Jingu
Keiji Nihei
**Keiji Suzuki**
Keiko Shibuya
Keizo Tano
Ken Ohnishi
Ken Yoshida
Kenichi Tanaka
Kenichi Yamada
Kenji Sekiguchi
Kenji Takayama
Kensuke Otsuka
Kenta Miwa
Kikuo Shimizu
Kimio Tanaka
Kohei Akazawa
Koichi Isobe
**Koji Tsuboi**
Kumiko Karasawa
Kunihiko Tateoka
Lakhan Kma
Lawrence Heibron
Lijun Wu
Lu Cai
Lu-Hua Wang
Makoto Shinoto
Marie Davidkova
Marjan Boerma
Masaaki Tatsuka
Masahiko Miura
Masahiko Oguchi
Masahiko Okumura
Masahiro Hosoda
Masahiro Kenjo
Masahiro Konno
Masanori Tomita
**Masao Suzuki**
**Masashi Chatani**
**Masataka Oita**
Masateru Ikehata
**Masatoshi Suzuki**
**Masayori Ishikawa**
Michel Drouet
**Michiaki Kai**
Miho Nemoto
Minako Sumi
Ming Zhao
Mingzhang Lin
Mitsuaki Ojima
Mitsuhiro Nakamura
Mohan Natarajan
Monica Olcina
Motohiro Yamauchi
Munetoshi Maeda
Naofumi Akata
Naoki Hayashi
Naoki Nakamura
Naomi Harley
Naoto Shikama
Naoya Shikazono
Naruhiro Matsufuji
Nasser H Hanna
**Natsuo Oya**
Natsuo Tomita
Nitin Gandhi
Noboru Takamura
Nobue Uchida
Nobuyoshi Fukumitsu
Norio Suzuki
Noriyuki Kadoya
Norman Kleiman
Osamu Inanami
Phil Jenkins
Piero Fossati
Qian Liu
R.K. Sachs
Rebecca Abergel
Rei Ogawa
Rikiya Onimaru
Robert Metzger
Robert Miller
Ryoichi Yoshimura
Ryota Washio
Ryouhei Yoshihara
Ryuichi Okayasu
Salwa Abdelkawi
Sanchita Ghosh
Satoru Monzen
Satoshi Fujii
Satoshi Ishikura
Satoshi Nakasono
Seiichi Wada
Selvakumar Anbalagan
Sheng-Lin Ma
Shigekazu Fukuda
Shin Koyama
Shin Toyoda
Shin-Ei Noda
Shingo Kato
Shinichi Shimizu
Shin-Ichiro Masunaga
**Shinichiro Mori**
Shinji Tokonami
Shinji Yoshinaga
Shinya Takemoto
Shubhadeep Purkayastha
Shuichi Kanamori
Shuichi Ozawa
Shunsuke Yonai
Shyam K. Shrivastava
Simon Cheng
Suguru Dobashi
Sumitaka Hasegawa
Sunao Tokumaru
Taiki Takaoka
Takafumi Toita
Takaharu Nomura
**Takahumi Komiyama**
Takashi Hanada
Takashi Kondo
Takashi Sato
Takashi Sugihara
**Takashi Uno**
Takashi Yagi
**Takeo Takahashi**
Takeshi Ebara
Takeshi Kamomae
Takeshi Kodaira
Takeshi Nishioka
Takeshi Todo
Takuma Nomiya
Takuyo Kozuka
Taro Murai
Tatsuhiko Imaoka
Tatsuhiko Sato
**Tatsuya Ohno**
Tetsuji Imanaka
Tetsuo Akimoto
Tetsuya Sakashita
Thomas Friedrich
Tohru Yamamori
Tokuhisa Hirouchi
Tomisato Miura
Tomoaki Tamaki
Tomohiko Yamane
Tomohiro Kaneta
Tomoko Kazumoto
Toru Takakura
Toshihiko Ozawa
Toshihiro Takatsuji
Toshio Mori
Toshiyasu Iwasaki
Toshiyuki Ogata
Toshiyuki Toshito
Tsuguhiro Miyashita
Tsukasa Aso
Vanessa Panettieri
Vincent Potiron
Wakatsuki Masaru
William F. Morgan
Yasuo Yoshioka
Yasuyuki Miyake
Yoichi Watanabe
**Yoko Harima**
Yoshihiko Manabe
**Yoshihiko Onizuka**
Yoshihiro Fujii
Yoshihiro Hase
Yoshihiro Namito
Yoshihiro Takai
Yoshihisa Matsumoto
Yoshikazu Nishimura
Yoshinori Sakurai
**Yoshitaka Matsumoto**
**Yoshiyuki Shioyama**
Yoshiyuki Suzuki
Ys Mayya
Yu Kumazaki
Yuichi Akino
Yuichi Michikawa
Yuichiro Yokota
Yuji Murakami
Yukari Yoshida
Yuki Miyabe
Yukihiro Hama
**Yukinori Matsuo**
**Yuko Kaneyasu**
Yuko Nakayama
Yurikusa Takashi
**Yuta Shibamoto**
Yutaka Takahashi
Yuu Ishimori
**Yuzuru Niibe**

